# The key role of Community Health Workers in the use of maternal services in rural settings: a Qualitative Study

**DOI:** 10.17843/rpmesp.2025.424.14898

**Published:** 2025-12-10

**Authors:** Fiorella Iturrino, Priscila Condori

**Affiliations:** 1 Charles University, Facultad de Políticas Públicas y Sociales, Praga, República Checa. Charles University Facultad de Políticas Públicas y Sociales Praga República Checa; 2 Pontificia Universidad Católica del Peru, Escuela de Gobierno y Políticas Públicas, Lima, Peru. Pontificia Universidad Católica del Peru Pontificia Universidad Católica del Perú Escuela de Gobierno y Políticas Públicas Lima Peru; 3 Erasmus University Rotterdam, International Institute of Social Studies, La Haya, Países Bajos. Erasmus Universiteit Rotterdam Erasmus University Rotterdam International Institute of Social Studies La Haya Netherlands

**Keywords:** Community Health Workers, Maternal Health, perception, Culturally Competent Care, Health Services, Qualitative Research

## Abstract

**Objective.:**

To understand the perception of the role of Community Health Workers (CHWs) as facilitators of the use of maternal health services in rural settings. Materials and methods. Semi-structured interviews were conducted with 11 CHWs assigned to the Paucara Health Center, selected by theoretical saturation sampling. Data were analyzed using a phenomenological approach, and constant comparison was used to inductively identify emerging themes.

**Results.:**

The interviews revealed three categories of perceptions: 1. Accompaniment and monitoring: pregnancy identification, home visits, community awareness, transfers in emergency cases, and challenges such as lack of remuneration and community misunderstandings; 2. Cultural and institutional mediation: integration of traditional practices, respectful treatment, addressing cultural barriers; 3. Management: reporting the situation of pregnant women, training, and communal referral.

**Conclusions.:**

CHWs perceive their roles as relevant in mitigating social, cultural, and structural barriers in maternal care. This study provides evidence for the design of public policies that can strengthen the recognition and sustainability of the CHWs’ work, through an equitable and culturally sensitive maternal care model.

## INTRODUCTION

Globally, approximately 700 women die every day from causes related to maternity ^1^; in Latin America, this figure rises to 30 deaths daily. In Peru, 263 maternal deaths were registered in 2023 ^2^, equivalent to one death per day. These figures show disparities in access to and use of maternal services ^3^, particularly in rural areas. In the latter, factors such as trust in the health system, language barriers, distance to facilities, resource availability, and limited infrastructure ^4^^-^^7^ constitute obstacles that restrict care. In regions like Huancavelica, where 69.5% of the population resides in rural areas and 39.5% ^8^ live in poverty, the gaps are more acute, suggesting the need for community interventions to improve maternal health ^9^. In this sense, Community Health Workers (CHWs) emerge as key intermediaries ^10^^,^^11^, linking communities with institutional health services ^12^.

In rural areas, culture plays a fundamental role in how women utilize maternal care services. In Paucara, Huancavelica, the Chopcca Campesina Community is characterized by its Andean cultural identity, with a Quechua-speaking population that is economically dependent on agriculture and livestock ^13^. The Andean cosmovision shapes a close relationship between individuals and their environment ^14^, establishing traditional maternal health practices based on principles of reciprocity and respect for community social norms ^15^^-^^17^. For the Chopcca woman, her traditional practices surrounding maternity involve the use of medicinal plants, vertical and squatting birth positions, the floor covered with animal hide, the attention of a traditional midwife (partera), and the presence of her relatives ^18^. However, the lack of integration of these practices into health facilities generates distrust and limits the use of maternal services ^19^.

Given this situation, women in rural areas require an intermediary to facilitate their access to and use of health services, helping to reduce the cultural distance from the institutional care system ^20^^,^^21^. This is how CHWs originated as a response to the scarcity of nearby health services and the difficulties rural communities face in using the services. In Peru, there are more than 35,000 CHWs ^22^, who extend their work to the areas furthest from higher-level health facilities, becoming a bridge between the health needs of the communities and the institutionalized services. Thus, their work is articulated and coordinated through local health facilities, which provide training and encourage collaboration so that the workers perform their duties effectively, with the aim of revaluing the traditional health system ^23^.

Although there is information on the documents that establish guidelines for the implementation of their actions and activities ^24^, their role has not been established based on a broader development of policies at the regional level ^25^. Furthermore, while there are investigations about CHWs as intermediaries, there is a scarcity of studies that explore their perceptions of their role in maternal health in Peruvian rural contexts, particularly in communities like the Chopcca, where community trust and cultural mediation are relevant. Therefore, the objective of this study is to understand the perceptions of CHWs regarding their roles as facilitators of maternal health service utilization in rural settings, particularly in Paucara, Huancavelica.

**KEY MESSAGES**

Motivation for the study. To understand the role of community health workers in the use of maternal services to overcome cultural, social, structural, and economic barriers faced by rural women.

Main findings. Community health workers perceive their roles in accompaniment and monitoring (pregnancy identification, visits, sensitization, transfers, challenges such as lack of remuneration), cultural and institutional mediation (traditional practices, respectful treatment), and management (reports, training, referrals), mitigating barriers in maternal care. In this way, they contribute to culturally appropriate maternal care.

Implications. The findings suggest the need to strengthen the role of community health workers to advance toward a culturally sensitive maternal care model.

## MATERIALS AND METHODS

### Study Design

This qualitative study has a phenomenological orientation, based on constant comparison to identify themes that reflect the perceptions of CHWs to establish meaningful categories inductively. Furthermore, it is based on semi-structured interviews, which allowed for an in-depth exploration of the perceptions and meanings participants assign to their social reality ^26^. According to Husserl ^27^, phenomenology seeks to understand the essence of phenomena and the lived experience in all its complexity, allowing meanings and awareness about these phenomena to emerge from the participants’ own perspective. In this sense, this approach is suitable for capturing the perceptions of CHWs, understood as meaningful constructions within a sociocultural context ^28^.

Moreover, this study was theoretically grounded in the WHO Social Determinants of Health framework ^29^, which allows understanding how individual, community, and structural circumstances influence the perceptions and practices of CHWs. In this sense, the methodology of the present study was selected based on the coherence of the phenomenological epistemological approach and the objective of the research. For this purpose, semi-structured interviews were the most appropriate instrument to capture a comprehensive view of individual experiences, facilitating the analysis of daily practices, sociocultural dynamics, and interactions between the actors investigated ^30^ and providing a broader context for understanding the social problems faced by the participants ^31^.

### Selection of Participants and Data Collection

Data collection was carried out based on a list of CHWs provided by the obstetrician in charge of interculturality at the Paucara Health Center. This obstetrician acts as a link between the CHWs and the institution through monthly meetings, and for this research, she played a key role in identifying and contacting the CHWs and the researchers. Since these actors are dispersed in rural communities with limited access to communication and transportation infrastructure, interviews were conducted by telephone. This modality allowed for the ethical participation of CHWs without affecting their daily activities, respecting their conditions of accessibility and connectivity, which in turn ensured the diversity of the sample and facilitated the collection of information.

The initial list included 48 names, of which 12 lacked a telephone number, and 16 had phones turned off or numbers out of service. The following inclusion criteria were applied to the remaining 20 CHWs: (i) being of legal age, (ii) having at least three years of experience as a CHW, which guaranteed accumulated knowledge about their work in the community, and (iii) agreeing to voluntarily participate in the study. Subsequently, 17 CHWs who met the established criteria were recruited. Theoretical saturation was reached with 11 participants, when the information gathered ceased to provide new data on the topics of interest ^32^. Following the recommendations of Saunders et al. ^33^, the research team evaluated theoretical saturation continuously and consistently with the study objective, the phenomenological approach, and the theoretical framework. An iterative analysis was applied in blocks of three interviews, and saturation was reached in the eleventh interview, when the narratives began to reiterate patterns of meaning; the remaining 6 participants confirmed the redundancy of the data. This procedure allowed for guaranteeing the interpretive depth of the study, ensuring the representation of the variability of the experiences and perspectives of the CHWs, in accordance with the principles of qualitative rigor ^34^.

The semi-structured interviews were conducted in January 2025 and had an average duration of 30 to 40 minutes. Given the logistical difficulties of applying a formal member checking, as a strategy that improves data validity and credibility ^35^, a specific verbal corroboration was implemented after each interview, based on an additional call to verbally confirm a summary of the responses. The latter allowed for immediate clarifications, although it does not constitute a procedure to ensure the total integrity of the information. The review of transcripts, or member checking, by participants is a strategy that improves data validity and credibility ^35^. In this study, it was adapted to contextual limitations due to barriers to technology access ^36^, which is why an additional call was made after the interviews to verbally confirm a summary of the responses. The latter allowed for maintaining the integrity of the information while respecting the accessibility and logistics of the participants.

### Data Analysis

Once the interviews were finalized, one of the researchers proceeded with the complete transcription of each one, ensuring that the processing accurately reflected the participants’ responses. The recordings were transcribed and organized entirely in the Obsidian software within 48 hours of their completion, ensuring accuracy in the documentation of the testimonies and minimizing potential loss of information. The transcription was done by FI and subsequently reviewed independently by PC, to ensure the accuracy and consistency of the analysis. The research team compared emerging versions to resolve discrepancies and guarantee the accuracy of the material.

The researchers performed the analysis using NVivo 14 software, in which an initial codebook was created based on a semi-structured interview guide prepared based on previous literature and study objectives, which is attached in Annex 1. Subsequently, the codes were grouped into categories, leading to the generation of three main themes, following the principles of thematic analysis by Braun and Clarke ^37^. To strengthen methodological rigor, strategies based on Salgado’s criteria ^34^ were applied: 1) Credibility: based on independent coding performed by PC, verbal verification, and cross-review; 2) Transferability: through the description of the context and selection by theoretical saturation; 3) Dependability: through systematic documentation of the data collection and analysis steps; and 4) Confirmability: through the record of analytical decisions and independent review of the transcripts. In this sense, the process of thematic analysis, developed in the coding and categorization, ensured that the interpretations emerged inductively from the data, in coherence with the phenomenological approach and the theory of Social Determinants of Health.

Likewise, the COREQ (Consolidated Criteria for Reporting Qualitative Research) guide was completed to ensure the standardization, transparency, and traceability of all methodological processes, which is attached in Annex 2. This document allowed for the clear and systematic documentation of all elements of the study, including participant selection, data collection, coding, and categorization of findings.

### Ethical Aspects

The study received ethical approval from the Research Ethics Committee of Charles University according to Act No. 157/2024 of November 22, 2024. This process ensured that the study activities were carried out following ethical principles and respecting the rights of the participants. The principles of justice, respect, and confidentiality were ensured following the ethical standards recommended for qualitative studies ^26^, considering the cultural and social context of rural communities.

To ensure justice, participant selection was performed using clear and transparent criteria defined in the “Selection of participants and data collection” subsection. In this study, the application of theoretical saturation allowed for including a sample that reflects the variability of CHWs’ perceptions within the framework of their work in the Paucara district. The principle of respect was guaranteed through the clear explanation of the study objective and procedures, as well as obtaining voluntary verbal informed consent. Participants’ right to withdraw at any time was emphasized. Furthermore, the interviews were adapted to the geographic and logistical conditions of the participants, by being conducted via telephone, which minimized interruptions to their activities.

To ensure participant confidentiality, confidentiality and privacy measures were implemented through the assignment of a unique identifying code. Likewise, secure storage of recordings and transcripts was carried out, which will be deleted in their entirety 12 months after the interviews are implemented. Finally, verbal verification of the summary of responses during a subsequent follow-up call allowed for ensuring the accuracy of the information without compromising participant privacy.

## RESULTS

The 11 participating CHWs are affiliated with the Paucara Health Center. Their age ranges between 35 and 65 years, with a predominance of those whose main economic activity is agriculture and livestock. On average, the interviewees have 19 years of experience working as CHWs in their communities. In terms of academic background, most have primary education and none have university studies, as detailed in Table 1.

**Table 1:** Sociodemographic Characteristics of CHWs

Variable		n = 11	%
Age			
	30 - 40	2	18.2
	40 - 50	4	36.4
	50 - 60	2	18.2
	more than 60 years	3	27.2
Level of education			
	No studies	2	18.2
	Completed primary education	3	27.3
	Incomplete primary education	2	18.2
	Completed secondary education	3	27.3
	Incomplete secondary education	1	9.1
Marital Status			
	Married	9	81.8
	Separated	2	18.2
Time dedicated to being a CHW			
	0 - 5 years	1	9.1
	5 - 10 years	1	9.1
	10 - 15 years	2	18.2
	More than 15 years	7	63.6
Main occupation*			
	Agriculture and Livestock	10	90.9
	Homemaker	1	9.1

Note*: refers to the main activity performed in addition to being a CHW

In the context of maternal health in rural settings, the CHWs described their perceptions of their roles, highlighting three categories: a) accompaniment and monitoring, b) cultural and institutional mediation, and c) management.

### Perception of the accompaniment and monitoring role

CHWs perceived their work as intermediaries between pregnant women and health services as essential to guarantee maternal care that aligns with the realities of rural communities. They highlighted that accompanying women throughout all stages of their pregnancy, childbirth, and puerperium is important, and in turn strengthens their adherence to prenatal services. Likewise, they described an active follow-up role through home visits, which allow for active surveillance. The latter ensures timely health care in risk situations, while they emphasize their promptness in identifying pregnant women and linking them to the health system:

*“[...] we are at the door, appearing, right away, we are running, and attending [...] (in) combination, we are there delivering (referring to childbirth), in health, the health post [...]”* (Interviewee 1)

*“[...] we help her (referring to the pregnant woman) when there is any accident, when there is anything, and we are there supporting her, taking her to the health post.”* (Interviewee 3)

The perception of their role in community sensitization about maternal health was relevant among the interviewees, who highlighted their effort to connect biomedical knowledge with traditional beliefs through educational and sensitization activities on maternal health topics, such as the importance of prenatal check-ups and risk prevention. They perceived that their work fosters the acceptance of health services by integrating local cultural beliefs, which generates changes in community attitudes toward institutional care.

*“Little by little we have sensitized, and people have slowly accepted the doctors because they didn’t even want the obstetricians to check their bodies, the pregnant women, for the medical check-up or for their check-ups, those things [...] sometimes we socialize by saying that the maternal home is like, it’s like your own home, there you can give birth however you prefer, sitting, lying down, there we have animal hides, there we have everything [...]”* (Interviewee 5).

Furthermore, CHWs perceived their role as persuasive, which reflects the commitment to managing the transfer of patients in cases of obstetric emergency, ensuring that women receive timely care in contexts of limited infrastructure, with scarcity of adequate transportation and poor road conditions:

*“[...] I also sensitized her to go to Acobamba so that she would necessarily go into the operating room to have the stitches removed, because the fluids were already running out, so I sensitized her and this month I already have a referral form from the community agent [...]”* (Interviewee 8).

*“[...] (before) it was difficult, there were no cars before, no motorcycles, nothing, and it was difficult. Now there are cars, motorcycles, they come faster now.”* (Interviewee 11)

In turn, a CHW highlighted the importance of dialoguing with families to prevent risks:

*“[...] and we talk to her partner, to her relatives, both her partner’s and the assistant’s, we talk to them, saying that, home birth cannot happen, because maternal death can occur, even perinatal death can occur [...]”* (Interviewee 8).

Despite perceiving a role of constant and relevant accompaniment and monitoring, CHWs referred to their work as a voluntary contribution that compensates for the limited presence of institutional health personnel in rural areas:

*“They, the ones who work at the health center, practically no longer visit, they no longer go, they no longer go out to the field. And we, as community agents, we truly work... there isn’t a single sol... we work voluntarily. [...] I am also a Quechua speaker and I like to speak more in Quechua, and we work like them, like the professionals who work at the health center, and we work without a salary, without anything.”* (Interviewee 10).

*“No, no, we don’t have anything, the municipality only gives us a small basket once a year.”* (Interviewee 2).

Even so, several CHWs mentioned that there is a perception in their communities that they receive remuneration for their work, which generates misunderstandings regarding the recognition they receive:

*“[...] without them paying us, it’s voluntary, that we participate, sometimes, it also happens, not here, but in other places too, señorita, it happens that sometimes they think we earn a salary, in reality no, we don’t earn, but now we have also always asked them to pay us (referring to the institutional managers).”* (Interviewee 3)

*“They tell me: ‘Surely, they are earning, they are earning, that’s why you earn. You wouldn’t waste your time.’ When in reality they don’t pay you, that’s what they told us.”* (Interviewee 9).

Collectively, CHWs perceived their monitoring and follow-up role as work of community commitment, where accompaniment, sensitization, and surveillance reinforce their identity as mediators, despite material limitations and the scarce recognition of their work.

### Perception of the cultural and institutional mediation role

CHWs perceived their role as cultural and institutional mediators in the context of maternal health in rural settings. They highlighted the integration of cultural practices into maternal care services, the promotion of respectful treatment by health personnel that generates dignified and empathetic care, the fostering of autonomy in pregnant women through addressing gender norms, linguistic barriers, and health infrastructure.

The incorporation of traditional practices and beliefs allows CHWs to facilitate the acceptance of maternal services by the women in their communities. They described the use of traditional herbs and techniques as part of their accompaniment for pregnant women. For CHWs, the use of traditional medicine allows for channeling women’s concerns toward health facilities, which ensures an approach that respects their cultural rights. One CHW mentioned:

*“[...] my wife (who is also a CHW) is respecting with herbs, with warm waters, we are here, the patient, we are tied with our scarves on their heads, with the chumpis, the cords [...]”* (Interviewee 1).

Another pointed out:

*“[...] for pregnant women, this, prepare this, what is it called, fish head broth, and also give them some herbs so that it doesn’t hurt while [...] in childbirth, and give them some herbs like that in water [...] so that the baby comes out smoothly and quickly. [...] you see, they help the pregnant woman a lot not to suffer in childbirth when giving birth to her child.”* (Interviewee 4).

Likewise, some CHWs recognize the implementation of cultural practices in health facilities.

*“But now, nowadays they believe (referring to cultural practices), now the health centers, some also ask, so they also train themselves from us [...] That’s how we work.”* (Interviewee 10).

In turn, CHWs described their role in fostering more respectful and empathetic treatment toward pregnant women by health personnel. Some CHWs addressed previous experiences of mistreatment or indifference, which have been overcome thanks to their work:

*“[...] but now they respect (referring to the Health Center staff), because we also pay attention to the doctors, because they have to be attentive, they have to be patient, it’s not for yelling. The women also already know their rights, what they are, how they can receive care at the health center, the women already know everything about their rights [...] Before it wasn’t (like that), they treated them badly, they complained (quenteaban, meaning complained) because they hadn’t come [...]”* (Interviewee 2).

*“[...] and when suspecting (a pregnancy) we have to inform the health center saying that such a person is pregnant. [...] And you have to have or gain more trust for them to tell you, for them to share. [...] sometimes one, sometimes they treat them badly [...] it already happened with me [...] When there are births and upon learning that they are minors, they ask their age, and at the same time treat them badly.”* (Interviewee 3).

*“[...] but at the health center they don’t attend right there, they make them wait like that. The pregnant mothers who are here, maybe they say, ‘they yelled at me’, that’s what they tell me, why would they yell at the women? [...] now they are already listening (referring to the women and their refusal to go to health centers). They only listen to us.”* (Interviewee 9).

Regarding gender norms and family power dynamics, CHWs perceived that their role includes promoting the autonomy of pregnant women through their active participation in decision-making regarding their pregnancy and childbirth. They believe that their work facilitates women’s capacity to decide about their own care, which can transform power dynamics within the home. However, gender barriers and the predominance of machismo still represent a structural challenge that limits the women of Paucara:

*“It’s just that before there was machismo, sometimes their husbands didn’t want them to go to the health center, and they just gave birth at home, because they had distrust. Now, almost due to training, we also, as community agents, because we provide training, they now trust us.”* (Interviewee 2).

*“[...] yes there are mothers who still sometimes the women who are from earlier times, from past eras, who are grandmothers or mothers-in-law, they don’t want the women to come to the health center, there is still machismo [...]”* (Interviewee 5)

A CHW described his work in addressing family violence:

*“All, all (CHWs) work on family violence, mental, psychological, physical abuse to everyone [...].”* (Interviewee 10)

However, the violence is not necessarily framed within the family space. Some CHWs mentioned the fear of pregnant women toward health personnel during institutional childbirth:

*“[...] some women or girls are afraid of being cut in the belly or of childbirth, when they can’t give birth, so they give birth in their homes, and then when they give birth, they go.”* (Interviewee 4)

*“Of course, there are women here, the pregnant women don’t want to go to the health center. Do you know why? Because sometimes when they give birth, then they enter, well, the room to be there. So there are also professionals. They are not conscious like that. They don’t feel like family. So they force the mothers, the women, to lie down on the bed. [...] They are afraid to go to the health center.”* (Interviewee 10)

Likewise, CHWs reported that their work facilitates communication between Quechua-speaking pregnant women and health personnel who only speak Spanish:

*“[...] they want me to speak to them in Quechua (during prenatal check-ups).”* (Interviewee 11)

*“[...] they come to the health center, there are others who speak Quechua [...], they don’t understand Quechua, and when they are here in Quechua they don’t pay attention to them [...]”* (Interviewee 6)

Some indicated that by being a communication bridge, they ensure effective and culturally relevant care, allowing pregnant women to understand their options and actively participate in key decisions, such as the choice between a vertical or horizontal birth, as one CHW highlights:

*“[...] at the health center, I tell them, there are vertical births and horizontal births, there are two, I speak to them in Quechua.”* (Interviewee 8)

Finally, CHWs perceived that their work extends to addressing structural and institutional demands, channeling infrastructure and equipment needs to the relevant authorities. In a context where rural health facilities present structural deficiencies and scarce equipment, CHWs considered it essential to make these shortcomings visible and promote institutional responses that guarantee better maternal care conditions. A CHW expressed:

*“Many things are missing. [...] The machines are missing, there aren’t any. Sometimes, for example, when we call the Health Center, we need an ambulance. Some don’t have fuel, they don’t have a driver. We get to the Health Center somehow. But like this, by motorcycle [...] somehow, we take them, we arrive with the families or with the neighbors, with the pregnant mothers who are there [...]”* (Interviewee 10)

The testimonies show that CHWs perceive themselves as key actors who integrate cultural traditions into maternity, language, and community participation with institutional care; in this way, they exercise a mediation role that goes beyond their community support.

### c) Perception of the management role

CHWs perceived their management role as fundamental to ensuring the continuity of maternal care in rural communities. Three main aspects were identified: the preparation and submission of reports on maternal health, participation in meetings and training sessions with health personnel, and the coordination of the referral process for pregnant women at risk.

Firstly, CHWs reported that they collect and transmit information about the pregnant women under their care to the Paucara Health Center; this includes monthly reports and records of home visits. Two CHWs described this process:

*“The person who attends to me gives me a receipt, what is it called? The card that I have from last year, and I will have about two or three there. Señorita, I forgot to deliver it to the health center, that’s why that time when we went to a meeting in December, I showed it to them and I brought it [...]”* (Interviewee 3)

*“[...] I have a little notebook, a home visit notebook, in it I make the home visit, and my signature, the pregnant woman’s signature and her fingerprint, I take that to the Paucara center [...] so this year I also commit [...] they gave a nice basket to all the most active promoters”* (Interviewee 8)

Meetings and training sessions with health personnel were activities described as opportunities to discuss particular cases of pregnant women in the communities and acquire knowledge about maternal health.

*“[...] precisely, now, mamita, we are going on Wednesday, there will even be a meeting at the municipality.”* (Interviewee 1)

*“[...] yes, we go monthly for training, we know what a danger sign is, what is a sign of labor, what are the alarm signs after labor [...]”* (Interviewee 3)

*“[...] every month, every fortnight [...] they give us training here at the Municipal Auditorium, or downstairs at the Paucara Health Center Auditorium [...]”* (Interviewee 8)

The training provided by health personnel has allowed CHWs to recognize the relevance of their management role in the referral process, which ensures the continuity of maternal care in cases of risk. They also reported coordinating the transfer of pregnant women with complications to the Paucara Health Center or higher-level facilities, completing referral forms to document the patients’ conditions. One of the CHWs described a specific case:

*“This month I already had a referral form from the community agent for a pregnant woman with four centimeters of dilation and I took her to the Paucara health center and from there she didn’t progress with her dilation and I convinced her, I talked to her relatives and she didn’t progress. She remained with four centimeters of dilation, with three or four centimeters of dilation, and they sent her to Acobamba (the province) [...]”* (Interviewee 8)

Another CHW expressed his support role:

*“[...] we also do the ‘replicas’ (referring to “referrals”) in the communities and with the pregnant mothers.” (Interviewee 5)*

Finally, CHWs perceived that their management role makes them key actors for the articulation between their communities and maternal health services. In the words of a CHW:

*“[...] we work supporting and we are the right hand of the Paucara Health Center and the workers, the nurses, and the obstetricians [...]”* (Interviewee 10)

The testimonies show the perception of the management role as an essential component of the work performed by CHWs. For them, their work is not limited only to collecting useful data for the Paucara Health Center, but also to guaranteeing the continuity of institutionalized maternal care, strengthening the relationship with health personnel, and ensuring that biomedical interventions are effective and culturally sensitive.

## DISCUSSION

This study aimed to understand the perceptions of Community Health Workers regarding their roles as facilitators of maternal health service utilization in the district of Paucara, Huancavelica. Based on a phenomenological analysis with constant comparison, the interview data allowed for identifying three relevant categories: accompaniment and monitoring, cultural and institutional mediation, and management. These categories were selected inductively and allowed for reflecting how CHWs perceive their work in addressing social determinants of health, such as cultural, gender, structural, and economic barriers.

Regarding accompaniment and monitoring, participants described their role as constant and active support for their communities, including early pregnancy identification, home visits, community sensitization, emergency transfers, and challenges associated with the lack of remuneration for their work and community misunderstandings regarding the recognition they receive. These perceptions align with studies that highlight the role of CHWs in improving and increasing the use of institutional maternal health services in rural settings ^25^^,^^38^^-^^41^. For example, the speed with which agents connect pregnant women and health centers *“[...] we are there supporting her, taking her to the health post [...]”* (Interviewee 3), and the perception of their work as voluntary *“[...] we don’t have anything, the municipality only gives us a small basket once a year”* (Interviewee 2), reflect their contribution to mitigating structural and economic barriers ^42^^,^^43^. However, this contribution is framed within a model of symbolic compensation that not only undervalues their contribution to public health but also imposes practical barriers to the sustainability of their work, especially in contexts of economic precariousness.

The interviews showed that CHWs perceive their role in cultural and institutional mediation as a bridge that integrates ancestral knowledge into maternal care, promotes respectful treatment, addresses gender barriers, and facilitates communication between health personnel and pregnant women. These perceptions resonate with studies regarding the role of CHWs as intercultural mediators ^44^^-^^46^. For example, the use of traditional medicine *“[...] my wife (who is also a CHW) is respecting with herbs [...]”* (Interviewee 1) and addressing machismo *“[...] sometimes their husbands didn’t want them to go to the health center [...], because we provide training, they now trust us.”* (Interviewee 2) highlight how CHWs contribute to mitigating cultural barriers ^47^^-^^50^. However, experiences of mistreatment *“So they force the mothers, the women, to lie down on the bed. [...]* They are afraid to go to the health center” (Interviewee 10) suggest the need for culturally more inclusive approaches ^17^, which allow for the coexistence and mutual benefit of traditional health and the biomedical system, in order to optimize maternal health outcomes in rural settings.

Likewise, the data evidenced that CHWs view their management role as essential for the continuity of maternal care, based on the training received, the reports, and the referrals they make to health centers. These perceptions align with studies on community-institutional articulation ^51^^-^^53^. In this sense, the coordination of *referrals “This month I already had a referral form from the community agent [...]” (Interviewee 8)* reflects their work in overcoming structural barriers that limit the use of maternal services in rural areas ^25^^,^^54^^,^^55^ from a key position *“[...] we work supporting and we are the right hand of the Paucara Health Center” (Interviewee 10).*

The perceptions of CHWs reveal an understanding of their role within maternal health care. They conceive of themselves as mediators and companions in the maternal process ^56^, rather than just facilitators of care. From this perspective, their identification as cultural bridges ^30^^,^^50^^,^^57^, and not merely as executors of maternal services, brings relevant implications for public health. When CHWs recognize their role as linguistic mediators, bridges of trust, and community promoters, their perceptions guide the way they contribute to improving the acceptance of services by pregnant mothers and biomedical knowledge. In public health, the study highlights the need for policies that strengthen the role of CHWs through strategies that ensure sustainable incentives such as continuous health training and adequate remuneration, in order to guarantee the permanence of their work. These incentives could contribute to maternal care that is more sensitive to the sociocultural contexts of rural populations.

This study presents limitations consistent with the transferability of results due to the use of theoretical saturation and semi-structured interviews, as well as the lack of data triangulation, the lack of elaboration of a code tree, and the lack of reflexivity on the role of the actors, which does not allow for generalization in rural settings in Peru.

However, the phenomenological approach used allowed for an understanding of the perceptions of CHWs, providing a contextualized perspective on their roles in Paucara.

In conclusion, this study shows that CHWs perceive their roles in accompaniment, monitoring, cultural and institutional mediation, and management as essential for facilitating the use of maternal health services in rural settings, addressing cultural, gender, structural, and economic barriers. These findings provide evidence for the design of public policies that can strengthen the recognition and sustainability of the work of Community Health Workers, through an equitable and culturally sensitive maternal care model.
