# Supplementary material for: The key role of Community Health Workers in the use of maternal services in rural settings: a Qualitative Study
Source: Rev Peru Med Exp Salud Publica. 2025 Dec 10;42(4):379–88. doi: 10.17843/rpmesp.2025.424.14898 (PMC12879984; doi:10.17843/rpmesp.2025.424.14898)
Supplement: Translation

# Translation: El rol clave de los Agentes Comunitarios de Salud en el uso de servicios maternos en entornos rurales: un estudio cualitativo

**Authors:** Fiorella Iturrino, Priscila Condori

**Affiliations:** 1 Charles University, Faculty of Public and Social Policies, Prague, Czech Republic.; 2 Pontificia Universidad Católica del Perú, School of Government and Public Policy, Lima, Perú.; 3 Erasmus University Rotterdam, International Institute of Social Studies, The Hague, Netherlands.

**Keywords:** Agentes Comunitarios de Salud, salud materna, percepción, Asistencia Sanitaria Culturalmente Competente, Servicios de Salud, Investigación Cualitativa

## Abstract

**Objetivo.:**

Comprender la percepción del rol de los Agentes Comunitarios de Salud (ACS) como facilitadores del uso de servicios de salud materna en entornos rurales.

**Materiales y métodos.:**

Se realizaron entrevistas semiestructuradas a 11 ACS asignados al Centro de Salud Paucará, seleccionados mediante muestreo por saturación teórica. Los datos se analizaron mediante un enfoque fenomenológico, y se utilizó una comparación constante para identificar los temas emergentes de manera inductiva.

**Resultados.:**

Las entrevistas revelaron tres categorías de percepciones: 1. Acompañamiento y monitoreo: identificación de embarazos, visitas domiciliarias, sensibilización comunitaria, traslados en casos de emergencia y desafíos como la falta de remuneración y malentendidos comunitarios; 2. Mediación cultural e institucional: integración de prácticas tradicionales, trato respetuoso, abordaje de barreras culturales; 3. Gestión: reporte de la situación de las gestantes, capacitación y referenciación comunal.

**Conclusiones.:**

Los ACS perciben sus roles como relevantes para mitigar barreras sociales, culturales y estructurales en la atención materna. Este estudio brinda evidencia para el diseño de políticas públicas que puedan fortalecer el reconocimiento y la sostenibilidad de la labor de los ACS, a través de un modelo de atención materna equitativo y culturalmente sensible.

## INTRODUCCIÓN

A nivel mundial cada día mueren alrededor de 700 mujeres por causas relacionadas con la maternidad ^(^^1^, en América Latina esta cifra asciende a 30 muertes diarias. En el Perú, en el 2023 se registraron 263 muertes maternas ^(^^2^, equivalente a una muerte por día. Estas cifras muestran disparidades en el acceso y uso de servicios maternos ^(^^3^, particularmente en zonas rurales. En estas últimas, factores como la confianza en el sistema de salud, las barreras lingüísticas, la distancia a los establecimientos, la disponibilidad de recursos y la infraestructura limitada ^(^^4^^-^^7^ constituyen obstáculos que restringen la atención. En regiones como Huancavelica, donde el 69,5% de la población reside en zonas rurales y el 39,5% ^8^ se encuentra en condición de pobreza, las brechas son más agudas, lo que sugiere la necesidad de intervenciones comunitarias para mejorar la salud materna ^(^^9^. En ese sentido, los Agentes Comunitarios de Salud (ACS) emergen como intermediarios clave ^(^^10^^,^^11^, vinculando a las comunidades con los servicios de salud institucional ^(^^12^.

En las zonas rurales, la cultura desempeña un papel fundamental en la forma en que las mujeres utilizan los servicios de atención materna. En Paucará, Huancavelica, la Comunidad Campesina Chopcca se caracteriza por su identidad cultural andina, con una población quechua hablante y dependiente económicamente de la agricultura y ganadería ^(^^13^. La cosmovisión andina configura una relación estrecha entre los individuos y su entorno ^(^^14^, estableciendo prácticas tradicionales de salud materna basadas en principios de reciprocidad y respeto por las normas sociales de la comunidad ^(^^15^^-^^17^. Para la mujer Chopcca, sus prácticas tradicionales entorno a la maternidad implican el uso de plantas medicinales, el parto vertical y de cuclillas, el suelo cubierto de pellejo de ganado, la atención de una partera y la presencia de sus familiares ^(^^18^. Sin embargo, la falta de integración de estas prácticas en los establecimientos de salud genera desconfianza y limita el uso de servicios maternos ^(^^19^.

Ante esta situación, las mujeres en zonas rurales requieren un intermediario que les facilite el acceso y uso de los servicios de salud, ayudando a reducir la distancia cultural con el sistema institucional de atención ^(^^20^^,^^21^. Es así como los ACS nacen como respuesta a la escasez de servicios de salud cercanos y a las dificultades que enfrentan las comunidades rurales para usar los servicios. En el Perú, existen más de 35,000 ACS ^(^^22^, quienes extienden su labor a las zonas más alejadas de los establecimientos de salud de mayor nivel, convirtiéndose en un puente entre las necesidades de salud de las comunidades y los servicios institucionalizados. De este modo, su trabajo se articula y coordina a través de los establecimientos de salud locales, los cuales brindan capacitación y fomentan la colaboración para que los agentes desempeñen sus funciones de manera efectiva, con el fin de revalorizar el sistema de salud tradicional ^(^^23^.

A pesar de que existe información sobre los documentos que establecen directrices para la implementación de sus acciones y actividades ^(^^24^, su rol no ha sido establecido a partir de un desarrollo más amplio de políticas a nivel regional ^(^^25^. Además, aunque existen investigaciones sobre los ACS como intermediarios, hay una escasez de estudios que exploren sus percepciones sobre su rol en la salud materna en contextos rurales peruanos, particularmente en comunidades como la Chopcca, donde la confianza comunitaria y la mediación cultural son relevantes. Por lo tanto, el objetivo de este estudio consiste en comprender la percepción del rol de los ACS como facilitadores del uso de servicios de salud materna en entornos rurales, particularmente en Paucará, Huancavelica.

**MENSAJE CLAVE**

Motivación para el estudio. Comprender el rol de los agentes comunitarios de salud en el uso de servicios maternos para superar las barreras culturales, sociales, estructurales y económicas de mujeres rurales.

Principales hallazgos. Los agentes comunitarios de salud perciben sus roles en acompañamiento y monitoreo (identificación de embarazos, visitas, sensibilización, traslados, desafíos como falta de remuneración), mediación cultural e institucional (prácticas tradicionales, trato respetuoso) y gestión (reportes, capacitación, referencias), mitigando barreras en la atención materna. De esa manera contribuyen a una atención materna culturalmente adecuada.

Implicancias. Los hallazgos sugieren la necesidad de fortalecer el rol de los agentes comunitarios de salud, para avanzar hacia un modelo de atención materna culturalmente sensible.

## MATERIALES Y MÉTODOS

### Diseño de estudio

Este estudio cualitativo es de orientación fenomenológica, basado en comparación constante para identificar temas que reflejan las percepciones de los ACS para establecer categorías significativas de manera inductiva. Asimismo, se fundamenta en entrevistas semiestructuradas, que permitieron explorar en profundidad las percepciones y significados que los participantes otorgan a su realidad social ^26^. Según Husserl ^(^^27^, la fenomenología busca comprender la esencia de los fenómenos y la experiencia vivida en toda su complejidad, permitiendo que los significados y la conciencia sobre dichos fenómenos surjan desde la propia perspectiva de los participantes. En ese sentido, este enfoque resulta adecuado para capturar las percepciones de los ACS, entendidas como construcciones significativas dentro de un contexto sociocultural ^(^^28^.

Asimismo, este estudio se fundamentó teóricamente en el marco de los Determinantes Sociales de Salud de la OMS ^(^^29^, que permite entender cómo las circunstancias individuales, comunitarias y estructurales influyen en las percepciones y prácticas de los ACS. En ese sentido, la metodología del presente estudio se seleccionó en función de la coherencia del enfoque epistemológico fenomenológico y el objetivo de la investigación. Para ello, las entrevistas semiestructuradas fueron el instrumento más adecuado para capturar una visión integral de las experiencias individuales, facilitando el análisis de las prácticas cotidianas, las dinámicas socioculturales y las interacciones entre los actores investigados ^30^^)^ y proporcionando un contexto más amplio para comprender los problemas sociales que enfrentan los participantes ^(^^31^.

### Selección de los participantes y recolección de información

La recopilación de datos se realizó a partir de un listado de ACS proporcionado por la obstetra encargada de interculturalidad del Centro de Salud Paucará. Esta obstetra actúa como nexo entre los ACS y la institución mediante reuniones mensuales, y para esta investigación desempeñó un papel clave en la identificación y el contacto con los ACS y las investigadoras. Dado que estos actores se encuentran dispersos en comunidades rurales con acceso limitado a infraestructura de comunicación y transporte, se optó por realizar las entrevistas de manera telefónica. Esta modalidad permitió garantizar la participación de los ACS de manera ética sin afectar sus actividades diarias, respetando sus condiciones de accesibilidad y conectividad, lo cual a su vez aseguró la diversidad de la muestra y facilitó la recopilación de información.

La lista inicial incluía 48 nombres, de los cuales 12 carecían de número telefónico, y 16 tenían teléfonos apagados o números fuera de línea. Para los 20 ACS restantes se aplicaron los siguientes criterios de inclusión: (i) ser mayor de edad, (ii) tener al menos tres años de experiencia como ACS, lo que garantizaba un conocimiento acumulado sobre su labor en la comunidad y (iii) aceptar participar voluntariamente en el estudio. Posteriormente se reclutó a 17 ACS que cumplían los criterios establecidos. La saturación teórica se alcanzó con 11 participantes, cuando la información recopilada dejó de aportar datos nuevos sobre los temas de interés ^(^^32^. Siguiendo las recomendaciones de Saunders *et al*. ^(^^33^, el equipo investigador evaluó la saturación teórica de manera continua y consistente con el objetivo del estudio, el enfoque fenomenológico y el marco teórico. Se aplicó un análisis iterativo en bloques de tres entrevistas, y la saturación se alcanzó en la undécima entrevista, cuando los relatos comenzaron a reiterar patrones de significado; los 6 participantes restantes confirmaron la redundancia de los datos. Este procedimiento permitió garantizar la profundidad interpretativa del estudio, asegurando la representación de la variabilidad de las experiencias y perspectivas de los ACS, de acuerdo con los principios del rigor cualitativo ^(^^34^.

Las entrevistas semi estructuradas se llevaron a cabo en enero de 2025 y tuvieron una duración promedio de 30 a 40 minutos. Dadas las dificultades logísticas de aplicar un member checking formal, como estrategia que permite mejorar la validez y credibilidad de los datos ^35^, se implementó una corroboración verbal puntual después de cada entrevista a partir de una llamada adicional para confirmar verbalmente un resumen de las respuestas. Esto último permitió aclaraciones inmediantes, aunque no constituye un procedimiento para asegurar la integridad total de la información. La revisión de transcripciones, o *member checking*, por parte de los participantes es una estrategia que permite mejorar la validez y credibilidad de los datos ^(^^35^. En este estudio se adaptó a las limitaciones contextuales debido a las barreras de acceso a la tecnología ^(^^36^, por lo cual después de las entrevistas se realizó una llamada adicional para confirmar verbalmente un resumen de las respuestas. Esto último permitió mantener la integridad de la información respetando la accesibilidad y logística de los participantes.

### Análisis de datos

Una vez finalizadas las entrevistas, una de las investigadoras procedió con la transcripción íntegra de cada una de ellas, asegurando que el procesamiento refleje con precisión las respuestas de los participantes. Las grabaciones fueron transcritas y organizadas en el software Obsidian en su totalidad dentro de las 48 horas posteriores a su realización, asegurando precisión en la documentación de los testimonios y minimizando potenciales pérdidas de información. La transcripción fue realizada por FI y posteriormente revisada de manera independiente por PC, para asegurar la exactitud y consistencia del análisis. El equipo investigador comparó versiones emergentes para resolver discrepancias y garantizar la exactitud del material.

Las investigadoras realizaron el análisis utilizando el software NVivo 14, en el que se creó un libro de códigos inicial basado en una guía de entrevista semiestructurada elaborada en función a literatura previa y objetivos del estudio, la misma que se adjunta en el Anexo 1. Posteriormente, los códigos se agruparon en categorías, dando lugar a la generación de tres temas principales, siguiendo los principios del análisis temático de Braun y Clarke ^(^^37^. A fin de fortalecer el rigor metodológico, se aplicaron estrategias basadas en los criterios de Salgado ^(^^34^: 1) Credibilidad: a partir de la codificación independiente realizada por PC, verificación verbal y revisión cruzada; 2) Transferibilidad: mediante la descripción del contexto y selección por saturación teórica; 3) Dependencia: a través de la documentación sistemática de los pasos de recolección y análisis; y 4) Confirmabilidad: mediante el registro de decisiones analíticas y revisión independiente de las transcripciones. En ese sentido, el proceso de análisis temático, desarrollado en la codificación y categorización, garantizó que las interpretaciones emergieran inductivamente de los datos, en coherencia con el enfoque fenomenológico y la teoría de los Determinantes Sociales de la Salud.

Asimismo, se completó la guía COREQ (Consolidated Criteria for Reporting Qualitative Research) para asegurar la estandarización, transparencia y trazabilidad de todos los procesos metodológicos, la misma que se adjunta en Anexo 2. Dicho documento permitió la documentación clara y sistemática de todos los elementos del estudio, incluyendo la selección de participantes, la recolección de datos, la codificación y la categorización de los hallazgos.

### Aspectos éticos

El estudio recibió aprobación ética del Comité de Ética en Investigación de la Charles University según el acta N° 157/2024 del 22 de noviembre de 2024. Este proceso garantizó que las actividades del estudio se lleven a cabo siguiendo principios éticos y respetando los derechos de los participantes. Se aseguraron los principios de justicia, respeto y confidencialidad siguiendo los estándares éticos recomendados para estudios cualitativos ^(^^26^, considerando el contexto cultural y social de las comunidades rurales.

Para asegurar la justicia, la selección de participantes se realizó mediante criterios claros y transparentes definidos en la subsección de Selección de participantes y recolección de información. En este estudio, la aplicación de la saturación teórica permitió incluir una muestra que refleje la variabilidad de percepciones de los ACS en el marco de su trabajo en el distrito de Paucará. El principio de respeto se garantizó mediante la explicación clara del objetivo y procedimientos del estudio, así como la obtención de consentimiento informado voluntario verbal. Se enfatizó el derecho de los participantes a retirarse en cualquier momento. Además, las entrevistas se adaptaron a condiciones geográficas y logísticas de los participantes, al ser realizadas vía telefónica, lo cual minimizó interrupciones en sus actividades.

A fin de asegurar la confidencialidad de los participantes, se implementaron medidas de confidencialidad y privacidad, mediante la asignación de un código único identificador. Asimismo, se realizó un almacenamiento seguro de las grabaciones y transcripciones, las mismas que serán eliminadas en su totalidad 12 meses después de la implementación de las entrevistas. Finalmente, la verificación verbal del resumen de las respuestas durante una llamada posterior de seguimiento permitió asegurar la exactitud de la información sin comprometer la privacidad de los participantes.

## RESULTADOS

Los 11 ACS participantes están adscritos al Centro de Salud Paucará. Su edad oscila entre los 35 y 65 años, predominando aquellos cuya actividad económica principal es la agricultura y la ganadería. En promedio, los entrevistados cuentan con 19 años de experiencia desempeñándose como ACS en sus comunidades. En términos de formación académica, la mayoría tiene educación primaria y ninguno posee estudios universitarios, como se detalla en la Tabla 1. Caracterización sociodemográfica de los ACS.

**Tabla 1:** Caracterización sociodemográfica de los ACS

Variable		n = 11	%
Edad			
	30 - 40	2	18,2
	40 - 50	4	36,4
	50 - 60	2	18,2
	más de 60 años	3	27,2
Nivel de escolaridad			
	Sin estudios	2	18,2
	Primaria completa	3	27,3
	Primaria incompleta	2	18,2
	Secundaria completa	3	27,3
	Secundaria incompleta	1	9,1
Estado civil			
	Casado(a)	9	81,8
	Separado(a)	2	18,2
Tiempo dedicado a ser ACS			
	0 - 5 años	1	9,1
	5 - 10 años	1	9,1
	10 - 15 años	2	18,2
	más de 15 años	7	63,6
Principal ocupación*			
	Agricultura y ganadería	10	90,9
	Ama de casa	1	9,1

Nota*: se refiere a la principal actividad que realiza además de ser ACS

En el contexto de salud materna en entornos rurales, los ACS describieron sus percepciones sobre sus roles destacando tres categorías: a) acompañamiento y monitoreo, b) mediación cultural e institucional y c) gestión.

### a) Percepción del rol de acompañamiento y monitoreo

Los ACS percibieron que su labor como intermediarios entre las gestantes y los servicios de salud es esencial para garantizar una atención materna que se ajuste a las medidas de las comunidades rurales. Destacaron que acompañar a las mujeres en todas las etapas de su embarazo, parto y puerperio resulta importante, y que a su vez fortalece la adherencia de estas a los servicios prenatales. Asimismo, describieron un rol activo de seguimiento a través de visitas domiciliarias, que permiten ejercer una vigilancia activa. Esto último asegura la atención oportuna de salud en situaciones de riesgo, en tanto resaltan su prontitud en identificación de gestantes y en vincularlas con el sistema de salud:

*“[...] estamos en la puerta, apareciendo, al toque, estamos corriendo, y atendiendo […] (en) conjunto, estamos ahí haciendo la luz (refiriéndose al parto), en la salud, la posta [...]”* (Entrevistado 1)

*“[...] nosotros la ayudamos (refiriéndose a la gestante) cuando hay cualquier accidente, cuando hay cualquier cosa y ahí estamos al lado apoyándole, llevándole a la posta.”* (Entrevistada 3)

La percepción sobre su rol de sensibilización comunitaria sobre salud materna fue relevante entre los entrevistados, quienes destacaron su esfuerzo en conectar conocimientos biomédicos con creencias tradicionales mediante actividades educativas y de sensibilización sobre temas de salud materna, como la importancia de los controles prenatales y la prevención de riesgos. Percibieron que su trabajo fomenta la aceptación de los servicios de salud al integrar creencias culturales locales, lo cual genera cambios en las actitudes de la comunidad hacia la atención institucional.

*“Poco a poco hemos sensibilizado y la gente ya poquito a poquito ha aceptado a los médicos porque ellos no querían ni que las obstetrices que le vieron su cuerpo las gestantes para la revisión médica o para sus controles, esas cosas [...] a veces nosotros sociabilizamos diciendo que el hogar materno es como, es como tu casa mismo, ahí puedes dar a luz como tú prefieras, sentada, echada, ahí tenemos pellejos, ahí tenemos de todo [...]”* (Entrevistada 5) 

Además, los ACS percibieron su rol como persuasivo, lo que refleja el compromiso con la gestión del traslado de pacientes en casos de emergencia obstétrica, asegurando que las mujeres reciban atención oportuna en contextos de infraestructura limitada, con escasez de transporte adecuado y precariedad vial: 

*“[...] le hice sensibilizar también para que vaya a Acobamba para que entre a sala de operación necesariamente para que saque a los hilitos, porque estaba ya por terminar los líquidos, entonces le hice sensibilizar y este mes ya tengo una hoja de referencia de agente comunitario [...]”* (Entrevistado 8)

*“[...] (antes) difícil era, no había carros anteriores, no había moto, nada y era difícil. Ahora hay carro, moto, vienen más rápido ya.”* (Entrevistado 11)

A su vez, un ACS destacó la importancia de dialogar con las familias para prevenir riesgos: 

*“[...] y lo hablamos a su pareja, a sus familiares, tanto de su pareja, tanto de la asistente, lo hablamos, es que, no puede ser parto domiciliario, porque puede pasar muerte materna, hasta muerte perinatal puede pasar [...]”* (Entrevistado 8)

A pesar de percibir un rol de acompañamiento y monitoreo constante y relevante, los ACS refirieron su trabajo como una contribución voluntaria que compensa la limitada presencia del personal de salud institucional en zonas rurales:

*“Ellos ya prácticamente los que trabajan en el centro de salud ya no visitan, ya no van, ya no salen al campo. Y nosotros como agente comunitario trabajamos de verdad…no hay ni un sol…trabajamos nosotros voluntariamente. [...] Yo soy quechua hablante también y me gusta hablar más en quechua y trabajamos como ellos, como los como profesionales que trabajan en el centro de salud y trabajamos nosotros sin sueldo, sin nada.”* (Entrevistado 10)

*“No no, no tenemos nada, solamente municipalidad nos da al año una canastita.”* (Entrevistada 2)

Aun así, varios ACS mencionaron que en sus comunidades existe la percepción de que reciben remuneración por su labor, lo que genera malentendidos respecto al reconocimiento que reciben:

*“[...] sin que nos paguen, es un voluntario, que nosotros participamos, a veces, siempre, también pasa, no será en aquí, pero en otros lugares también, señorita, pasa que a veces piensan ellos que nosotros ganamos, en realidad no no ganamos, pero ahora nosotros también hemos pedido siempre que nos paguen (refiriéndose a los responsables institucionales).”* (Entrevistada 3)

*“Ellos me dicen: ‘Ganando seguro, andarán, ganando, por eso ustedes ganan. No van a perder su tiempo’. Cuando en realidad no te pagan, así nos han dicho.”* (Entrevistada 9)

En conjunto, los ACS percibieron su rol de monitoreo y seguimiento como una labor de compromiso comunitario, donde el acompañamiento, la sensibilización y la vigilancia refuerzan su identidad como mediadores, a pesar de las limitaciones materiales y del escaso reconocimiento de su trabajo.

### b) Percepción del rol de mediación cultural e institucional

Los ACS percibieron su rol como mediadores culturales e institucionales en el contexto de la salud materna en entornos rurales. Destacaron la integración de prácticas culturales en los servicios de atención materna, la promoción de un trato respetuoso por parte del personal de salud que genera un trato digno y empático, el fomento de la autonomía en las gestantes mediante un abordaje de las normas de género, barreras lingüísticas y de infraestructura sanitaria.

La incorporación de prácticas y creencias tradicionales permite a los ACS facilitar la aceptación de los servicios maternos por parte de las mujeres de sus comunidades. Describieron el uso de hierbas y técnicas tradicionales como parte de su acompañamiento a las gestantes. Para los ACS el uso de la medicina tradicional permite canalizar las preocupaciones de las mujeres hacia los establecimientos de salud, lo cual garantiza un enfoque que respete sus derechos culturales. Un ACS mencionó:

*“[...] mi esposa (que también es ACS) está respetando con las hierbas, con las agüitas calientes, estamos acá, la paciente, estamos amarrados con sus pañuelitos las cabecitas, con las chumpis, los cordones [...]”* (Entrevistado 1).

Otro señaló:

*“[...] para las señoras que están embarazadas, este, prepara este, como se llama, caldo de pescado de cabeza, y también le da unas hierbas para que no pueda dolerle estando […] el parto, y le da unas hierbas así en agua [...] para que el bebé salga suave y rápido. [...] a la gestante la ayudan bastante, tú ves, a no sufrir en el parto dando su hijo.”* (Entrevistada 4).

Asimismo, algunos ACS reconocen la implementación de prácticas culturales en los establecimientos de salud.

*“Pero ahora, hoy en día ya creen (refiriéndose a las prácticas culturales), ya los centros de salud, algunos ya preguntan también, entonces ellos también se capacitan de nosotros [...] De esa manera trabajamos.” (Entrevistado 10).*

A su vez, los ACS describieron su rol en fomentar un trato más respetuoso y empático por parte del personal de salud hacia las gestantes. Algunos ACS abordaron experiencias de maltrato o indiferencia previos, los mismos que han sido superados gracias a su labor:

*“[...] pero ahora ya respetan (refiriéndose al personal del centro de Salud), porque nosotros también tomamos atención a los médicos, porque tienen que tener atención, tienen que estar con paciencia, no es para estar gritando. También las señoras ya saben sus derechos, qué son, cómo pueden tener atención en el centro de salud, todo ya saben las señoras sus derechos [...] Antes no era (así), trataban mal, quenteaban (quejaban), porque no han venido [...]”* (Entrevistada 2).

*“[...] y al sospechar (sobre un embarazo) ya tenemos que avisarle al centro de salud diciendo que tal persona está gestante. […] Y tienes que tener o ganar más confianza para que te avise, para que te cuente. [...] a veces uno, a veces la tratan mal […] hasta conmigo ya pasó ya […] Cuando hay partos y al saber que son ellas, son menor de edad, le preguntan su edad, y a la vez le tratan mal.”* (Entrevistada 3).

*“[...] pero en el centro de salud no atienden ahí mismo, les hacen demorar así. A las madres gestantes que están aquí, tal vez dicen, ‘me han gritado’, así me dicen, ¿por qué me van a gritar a las señoras? [...] ahora ya están escuchando ya (en referencia a las mujeres y su negativa para ir a los centros de salud). A nosotros nomás nos hacen caso.”* (Entrevistado 9).

Respecto a las normas de género y la dinámica de poder familiar, los ACS percibieron que su rol incluye promover la autonomía de las mujeres gestantes a través de su participación activa en la toma de decisiones respecto a su embarazo y parto. Ellos consideran que su labor facilita la capacidad de estas para decidir sobre su propio cuidado, lo cual puede transformar las dinámicas de poder dentro del hogar. Sin embargo, las barreras de género y el predominio del machismo aún representan un desafío estructural que limita a las mujeres de Paucará:

“*Es que más antes eran machismos, sus esposos a veces no querían que vayan al centro de salud, y en su casa nomás daban partos, por ahí tenían desconfianza. Ahora casi por capacitaciones, nosotros también como agentes comunitarios, porque damos capacitaciones, ya tienen confianza con nosotros.”* (Entrevistada 2).

*“[...] sí hay mamás que todavía a veces las señoras que son de anteriores tiempos, de épocas atrás, que son abuelas o suegras, ellos no quieren que vengan al centro de salud, las mujeres, que todavía hay machismo[...]”* (Entrevistada 5).

Un ACS describió su trabajo en abordar violencia familiar: 

*“Todos, todos (los ACS) trabajamos violencia familiar, mentalmente, psicológico, maltratos físicamente a todos [...].”* (Entrevistado 10).

Sin embargo, la violencia no necesariamente se enmarca en el espacio familiar. Algunos ACS mencionaron el temor de las gestantes hacia el personal de salud durante el parto institucional:

*“[...] algunas señoras o chicas tienen miedo a que le corten la barriga o al parto, así cuando no pueden dar a luz, entonces en sus casas dan a luz, y así cuando dan a luz ya, ya van.”* (Entrevistada 4).

*“Claro, hay mujeres acá, las gestantes no quieren ir al centro salud. ¿Sabe por qué? Porque a veces cuando dan a la luz, ya entonces ya entran, pues a la sala para que estén ahí. Entonces hay profesionales también. No son así conscientes. No sienten como su familia. Entonces le obligan a las mamitas, a las señoras que se metan a la camilla. [...] Tienen miedo de ir al centro de salud.”* (Entrevistado 10).

Asimismo, los ACS reportaron que su labor facilita la comunicación entre las gestantes que hablan Quechua y el personal de salud que únicamente habla Castellano:

*“[...] en Quechua, quieren que les hable (durante los controles prenatales).”* (Entrevistada 11).

*“[...] vienen al centro de salud, hay otros que hablan quechua [...], no comprenden quechua, y cuando está aquí en quechua no le hace caso[...]”* (Entrevistada 6).

Algunos indicaron que al ser ellos un puente de comunicación, garantizan una atención efectiva y culturalmente pertinente, permitiendo que las gestantes comprendan sus opciones y participen activamente en decisiones clave, como la elección entre un parto vertical u horizontal, así como destaca un ACS: 

*“[...] en el centro de salud, le digo, hay partos verticales y partos horizontales, hay dos, en Quechua le hablo.”* (Entrevistado 8).

Finalmente, los ACS percibieron que su labor se extiende a atender demandas estructurales e institucionales, canalizando necesidades de infraestructura y equipamiento hacia las autoridades pertinentes. En un contexto donde los establecimientos de salud rurales presentan deficiencias estructurales y escaso equipamiento, los ACS consideraron indispensable la visibilización de estas carencias y la promoción de respuestas institucionales que garanticen mejores condiciones de atención materna. Un ACS expresó:

*“Faltan muchas cosas. Faltan […] las máquinas, no hay. A veces, por ejemplo, así cuando llamamos también a Centro Salud, ya necesitamos ambulancia. Algunos no hay combustible, no hay chofer. Llegan al Centro Salud como sea nosotros. Pero así con moto […] como sea, llevamos, llegamos con las familias o con los vecinos, con las madres gestantes que están ahí [...]”* (Entrevistado 10).

Los testimonios muestran que los ACS se perciben como actores clave que integran las tradiciones culturales a la maternidad, la lengua y la participación comunitaria con la atención institucional, de esa manera, ejercen un rol de mediación que va más allá de su apoyo comunitario.

### c) Percepción del rol de gestión

Los ACS percibieron su rol de gestión como fundamental para garantizar la continuidad de la atención materna en comunidades rurales. Se identificaron tres aspectos principales: la elaboración y entrega de reportes sobre la salud materna, la participación en reuniones y capacitaciones con el personal de salud, y la coordinación del proceso de referencia para gestantes con riesgos.

En primer lugar, los ACS reportaron que recopilan y transmiten información sobre las gestantes a su cargo al Centro de Salud Paucará; esto incluye reportes mensuales y registros de visitas domiciliarias. Dos ACS describieron este proceso:

*“La persona que me atiende, me da un recibito, ¿cómo se llama? La tarjeta que yo tengo como de este año pasado, y tendré como dos o tres por ahí. A mi señorita me ha faltado entregarle al centro de salud, por eso yo esa vez cuando fuimos a reunión en diciembre, yo le he enseñado y yo he cargado [...]”* (Entrevistada 3).

*“[…] tengo un cuadernito, un cuaderno de visita domiciliaria, en ahí le hago visita domiciliaria, y mi firma, la gestante, su firma y su huella, eso lo llevo al centro de Paucará [...] entonces este año también me comprometo [...] me ha dado una buena canasta a todos los promotores más activos”* (Entrevistado 8).

Las reuniones y capacitaciones con el personal de salud fueron actividades descritas como oportunidades para discutir casos particulares de las gestantes de las comunidades y adquirir conocimientos de salud materna.

*“[...] justamente, ahora, mamita, estamos yendo el miércoles, va a haber hasta reunión en el municipio.”* (Entrevistado 1).

*“[...] sí, vamos mensual a capacitaciones, sabemos qué es signo de peligro, qué es del parto, después del parto, cuáles son los signos de alarma [...]”* (Entrevistada 3).

*“[...] cada mes, cada quincena […] nos dan capacitación aquí en el Auditorio Municipal, o abajo en el Auditorio del Centro de Salud Paucará […]”* (Entrevistado 8).

Las capacitaciones brindadas por el personal de salud han permitido que los ACS reconozcan la relevancia de su rol de gestión en el proceso de referencia, el cual garantiza la continuidad de la atención materna en casos de riesgo. Asimismo, reportaron que coordinan el traslado de gestantes con complicaciones hacia el Centro de Salud Paucará o a establecimientos de mayor nivel, completando hojas de referencia para documentar las condiciones de las pacientes. Uno de los ACS describió un caso específico:

*“Este mes ya tuve una hoja de referencia de agente comunitario a una gestante con cuatro dilataciones y he llevado al centro salud Paucará y de ahí no ha avanzado con su dilatación y le he hecho convencer, le he hablado con sus familiares y no ha avanzado. Se quedó con cuatro dilataciones, con tres o cuatro dilataciones se quedó y mandó a Acobamba (la provincia) [...]”* (Entrevistado 8).

Otro ACS manifestó su rol de apoyo:

“[...] nosotros también, hacemos las réplicas (se refiere a “referencias”) en las comunidades y con las madres gestantes.” (Entrevistada 5).

Finalmente, los ACS percibieron que su rol de gestión los convierte en actores clave para la articulación entre sus comunidades y los servicios de salud materna. En palabras de un ACS:

*“[...] trabajamos apoyando y somos su mano derecha del Centro Salud Paucará y de los trabajadores, de las enfermeras y de las obstetras [...]” (Entrevistado 10).*

Los testimonios muestran la percepción del rol de gestión como un componente esencial de la labor que cumplen los ACS. Para ellos, su trabajo no se reduce únicamente a la recopilación de datos útiles para el Centro de Salud Paucará, sino también a garantizar la continuidad de la atención materna institucionalizada, fortalecer la relación con el personal de salud y asegurar que las intervenciones biomédicas sean eficaces y culturalmente sensibles.

## DISCUSIÓN

Este estudio tuvo por objetivo comprender las percepciones de los Agentes Comunitarios de Salud respecto a sus roles como facilitadores del uso de servicios de salud materna en el distrito de Paucará, Huancavelica. A partir de un análisis fenomenológico con comparación constante, los datos de las entrevistas permitieron identificar tres categorías relevantes: acompañamiento y monitoreo, mediación cultural e institucional, y gestión. Estas categorías fueron seleccionadas inductivamente y permitieron reflejar de qué manera los ACS perciben su labor al abordar determinantes sociales de salud, como barreras culturales, de género, estructurales y económicas.

Respecto al acompañamiento y monitoreo, los participantes describieron su rol como un apoyo constante y activo hacia sus comunidades, que incluye la identificación temprana de embarazos, visitas domiciliarias, sensibilización comunitaria, traslados en casos de emergencia y desafíos asociados a la falta de remuneración por su labor y a malentendidos comunales respecto al reconocimiento que reciben. Estas percepciones se alinean con estudios que destacan el papel que cumplen los ACS en el mejoramiento e incremento de uso de servicios de salud materna institucionales en entornos rurales ^(^^25^^,^^38^^-^^41^. Por ejemplo, la rapidez con la que los agentes conectan a las gestantes y a los centros de salud *“[...] ahí estamos al lado apoyándole, llevándole a la posta [...]” (Entrevistada 3)*, y la percepción de su trabajo como voluntario *“[...] no tenemos nada, solamente municipalidad nos da al año una canastita” (Entrevistada 2)*, reflejan su contribución a mitigar barreras estructurales y económicas ^(^^42^^,^^43^. Sin embargo, esta contribución se enmarca en un modelo de compensación simbólica que no solo desvaloriza su aporte a la salud pública, sino que también impone barreras prácticas a la sostenibilidad de su trabajo, especialmente en contextos de precariedad económica.

Las entrevistas mostraron que los ACS perciben su rol de mediación cultural e institucional como un puente que integra conocimientos ancestrales en la atención materna, promueve un trato respetuoso, aborda barreras de género y facilita la comunicación entre el personal de salud y las gestantes. Estas percepciones resuenan con estudios respecto al rol de los ACS como mediadores interculturales^44^^-^^46^. Por ejemplo, el uso de medicina tradicional *“[...] mi esposa (que también es ACS) está respetando con las hierbas [...]” (Entrevistado 1)* y el abordaje del machismo *“[...] sus esposos a veces no querían que vayan al centro de salud [...], porque damos capacitaciones, ya tienen confianza con nosotros.” (Entrevistada 2)* destacan cómo los ACS contribuyen a mitigar barreras culturales ^(^^47^^-^^50^. Sin embargo, las experiencias de maltrato *“Entonces le obligan a las mamitas, a las señoras que se metan a la camilla. [...] Tienen miedo de ir al centro de salud” (Entrevistado 10)* sugieren la necesidad de enfoques culturalmente más inclusivos ^(^^17^, que permitan la coexistencia y el aprovechamiento mutuo de la salud tradicional y el sistema biomédico, a fin de optimizar los resultados en salud materna en entornos rurales.

Asimismo, los datos evidenciaron que los ACS ven su rol de gestión como esencial para la continuidad de la atención materna, a partir de las capacitaciones recibidas, los reportes y las referencias que realizan a los centros de salud. Estas percepciones se alinean con estudios sobre la articulación comunitaria-institucional ^(^^51^^-^^53^. En ese sentido, la coordinación de referencias *“Este mes ya tuve una hoja de referencia de agente comunitario [...]” (Entrevistado 8)* refleja su labor en superar barreras estructurales, que limitan el uso de servicios maternos en zonas rurales ^25^^,^^54^^,^^55^ desde una postura clave *“[...] trabajamos apoyando y somos su mano derecha del Centro Salud Paucará” (Entrevistado 10).*

Las percepciones de los ACS revelan un entendimiento de su rol dentro de los cuidados de salud materna. Ellos se conciben a sí mismos como mediadores y acompañantes del proceso materno^56^, más que únicamente facilitadores de atención. Desde esta perspectiva, su identificación como puentes culturales ^30^^,^^50^^,^^57^, y no únicamente como ejecutores del servicio materno, aporta implicancias relevantes para la salud pública. Cuando los ACS reconocen su rol como mediadores lingüísticos, puentes de confianza, y promotores comunitarios, sus percepciones orientan la forma en la que contribuyen a mejorar la aceptación de los servicios por parte de las madres gestantes y los saberes biomédicos. En la salud pública, el estudio destaca la necesidad de políticas que fortalezcan el rol de los ACS mediante estrategias que aseguren incentivos sostenibles como la formación continua en salud y una remuneración adecuada, a fin de que se garantice la permanencia de sus labores. Estos incentivos podrían contribuir a una atención materna más sensible a los contextos socioculturales de las poblaciones rurales.

Este estudio presenta limitaciones consistentes con la transferencia de resultados por uso de saturación teórica y entrevistas semiestructuradas, así como también la falta de triangulación de datos, la no elaboración de un árbol de códigos y la falta de reflexibidad del rol de los actores, lo cual no permite la generalización en entornos rurales del Perú.

No obstante, el enfoque fenomenológico empleado permitió una comprensión de las percepciones de los ACS, aportando una perspectiva contextualizada sobre sus roles en Paucará.

En conclusión, este estudio muestra que los ACS perciben sus roles de acompañamiento, monitoreo, mediación cultural e institucional y gestión como esenciales para facilitar el uso de servicios de salud materna en entornos rurales, abordando barreras culturales, de género, estructurales y económicas. Estos hallazgos brindan evidencia para el diseño de políticas públicas que puedan fortalecer el reconocimiento y la sostenibilidad de la labor de los Agentes Comunitarios de Salud, a través de un modelo de atención materna equitativo y culturalmente sensible.
